# Chloroquine-Resistant *Plasmodium* *vivax*, Brazilian Amazon

**DOI:** 10.3201/eid1307.061386

**Published:** 2007-07

**Authors:** Franklin Simoes de Santana Filho, Ana Ruth de Lima Arcanjo, Yonne Melo Chehuan, Monica Regina Costa, Flor Ernestina Martinez-Espinosa, Jose Luis Vieira, Maria das Graças Vale Barbosa, Wilson Duarte Alecrim, Maria das Graças Costa Alecrim

**Affiliations:** * Foundation for Tropical Medicine of Amazonas, Manaus, Amazonas, Brazil; † Foundation for Research Support of Amazonas, Manaus, Amazonas, Brazil; ‡ Federal University of Pará, Belém, Pará, Brazil; § Amazonas State University, Manaus, Amazonas, Brazil; ¶ Nilton Lins University, Manaus, Amazonas, Brazil

**Keywords:** malaria, Plasmodium vivax, resistance, antimalarial, chloroquine, Amazon, Brazil, Manaus, therapeutic failure, letter

**To the Editor:** *Plasmodium vivax* is the protozoan that causes the second most common form of malaria. Some resistant strains to chloroquine (CQ) occur in a few places in Asia and the Indo-Pacific Region (*1*–*4*). Although resistance of *P. vivax* to CQ has already been described in South America (*5*–*7*), there are limited data regarding this issue.

CQ plus primaquine is the standard treatment for vivax malaria worldwide. Presently, this drug regimen exhibits satisfactory efficacy in the Brazilian Amazon. However, in recent years several treatment failures presumably related to CQ resistance*,* have been reported in the city of Manaus (Amazonas) where vivax malaria predominates (*7*). This observation warrants local attention despite these cases having no confirmation of CQ blood levels on the basis of the appearance of asexual parasites against CQ plus desethylchloroquine levels exceeding the minimally effective plasma concentration proposed for sensitive parasite strains (>10 ng/mL) (*8*), according to Pan American Health Organization recommendations (*9*).

From September 2004 to February 2005, a 28-day in vivo test was conducted at the Foundation for Tropical Medicine of Amazonas (FMTAM) in Manaus, Brazil, to assess the efficacy of standard supervised CQ therapy. The test involved 166 volunteers with uncomplicated vivax malaria. Each volunteer was administered uncoated, scored, 150-mg CQ tablets (10 + 7.5 + 7.5 mg/kg at 24-hour intervals) (*9*). Primaquine was withheld until day 28 (dose regimen of 30 mg/day for 7 days). Among the 109 volunteers who completed the in vivo test, 19 had positive blood smears within the 28-day follow-up (1 on day 14, 3 on day 21, and 15 on day 28). All were required to undergo alternative therapy (mefloquine). Adequate CQ absorption was confirmed in these cases on day 2 with a mean ± SD CQ plasma concentration of 785.4 ± 800.1 ng/mL) (*10*) Suspected therapeutic failure (*P. vivax* CQ resistance) was confirmed in 11 (10.1%) of 109 persons with a mean isolated choloroquine plasma concentration >10 ng/mL (356.6 ± 296.1 ng/mL) (*9*). Desethylchloroquine levels in plasma were not measured.

Previously, a CQ efficacy study demonstrated that 4.4% of those tested had CQ-resistant *P. vivax* (*7*)*.* In comparison, the proportion of failures (10.1%) in the current study seems to be relevant; even though most of the *P. vivax* infections (98, 89.9%) were successfully evaluated and adequate clinical and parasitologic responses were obtained. Currently, the FMTAM Manaus Outpatient Clinic is detecting patients from different areas of the city who show parasitologic recurrences after correct treatment within 28 days of the routine clinical follow-up. This observation is an indirect indicator of the possible regional spread of *P. vivax* CQ-resistant strains (unpub. data).

We believe our findings are important and merit the attention of local public health authorities. Considering the possibility of emerging underestimated *P. vivax* CQ resistance in Manaus, we feel it is essential to quickly clarify whether such documented resistance can copromote vivax malaria outbreaks in malaria-endemic areas within the Amazon.
